# Data-driven decision making for the screening of cognitive impairment in primary care: a machine learning approach using data from the ELSA-Brasil study

**DOI:** 10.1590/1414-431X2023e12475

**Published:** 2023-01-27

**Authors:** C. Szlejf, A.F.M. Batista, L. Bertola, P.A. Lotufo, I.M. Benseãor, A.D.P. Chiavegatto, C.K. Suemoto

**Affiliations:** 1 Centro de Pesquisa Clínica e Epidemiológica, Hospital Universitário, Universidade de São Paulo, São Paulo, SP, Brasil; 2 Hospital Israelita Albert Einstein, São Paulo, SP, Brasil; 3 Departmento de Epidemiologia, Faculdade de Saúde Pública, Universidade de São Paulo, São Paulo, SP, Brasil; 4 Insper Instituto de Ensino e Pesquisa, São Paulo, SP, Brasil; 5 Divisão de Geriatria, Faculdade de Medicina, Universidade de São Paulo, São Paulo, SP, Brasil

**Keywords:** Artificial intelligence, Cognition, Prediction, Primary care

## Abstract

The systematic assessment of cognitive performance of older people without cognitive complaints is controversial and unfeasible. Identifying individuals at higher risk of cognitive impairment could optimize resource allocation. We aimed to develop and test machine learning models to predict cognitive impairment using variables obtainable in primary care settings. In this cross-sectional study, we included 8,291 participants of the baseline assessment of the ELSA-Brasil study, who were aged between 50 and 74 years and were free of dementia. Cognitive performance was assessed with a neuropsychological battery and cognitive impairment was defined as global cognitive z-score below 2 standard deviations. Variables used as input to the prediction models included demographics, social determinants, clinical conditions, family history, lifestyle, and laboratory tests. We developed machine learning models using logistic regression, neural networks, and gradient boosted trees. Participants' mean age was 58.3±6.2 years, 55% were female. Cognitive impairment was present in 328 individuals (4%). Machine learning algorithms presented fair to good discrimination (areas under the ROC curve between 0.801 and 0.873). Extreme Gradient Boosting presented the highest discrimination, high specificity (97%), and negative predictive value (97%). Seventy-six percent of the individuals with cognitive impairment were included among the highest ranked individuals by this algorithm. In conclusion, we developed and tested a machine learning model to predict cognitive impairment based on primary care data that presented good discrimination and high specificity. These characteristics could support the detection of patients who would not benefit from cognitive assessment, facilitating the allocation of human and economic resources.

## Introduction

According to the Global Burden of Diseases 2019, dementia currently accounts for 1% of the total disability-adjusted life years worldwide (1). Although this burden is still lower in low- and middle-income countries (LMIC) (1), the number of dementia patients in these countries is increasing faster than in high-income countries, raising a red flag for policy makers (2). In clinical practice, cognitive assessment is usually symptom-driven and based on cognitive complaints. Although some agencies recommend early detection of dementia in older adults (3,4), the systematic screening of asymptomatic individuals is controversial due to uncertainties regarding clinically meaningful effects and benefits of current dementia therapies, and regarding the interventions targeting caregiver burden and decision-making by patients, caregivers, and family (5,6). Moreover, in LMIC, time constraints and limited resources and training are barriers to cognitive assessment in primary care, precluding the implementation of screening programs.

Several algorithms have been developed to predict dementia in different populations, such as mid- and late-life risk models that use classical statistical analysis or machine learning (ML) (7,8). However, few of the existing predictive models rely on information that is obtainable in daily clinical practice and could be implemented in primary care settings, without requiring extensive neuropsychological batteries, neuroimaging, or genomics (9-13). Moreover, the literature is scarce on models developed to predict cognitive impairment based on data available in primary care prior to cognitive screening. Reijmer et al. (14) found that a model based on age, gender, education, systolic blood pressure, body mass index, and total cholesterol predicted future impairment in some cognitive domains in middle-aged adults. A ML model was developed in South Korea to predict future cognitive impairment in older adults based on sociodemographic factors, health conditions, functional ability, and subjective well-being (15).

As cognitive impairment results from the interaction of biological, behavioral, and social processes throughout life (2), it is a challenge to develop accurate predictive models based on information obtainable in the primary care routine. ML, a branch of artificial intelligence and computer science, uses statistical algorithms that learn patterns from non-linear, high-dimensional data and consider interactions between multiple predictors (16). Therefore, we aimed to develop ML models to predict cognitive impairment in middle-aged and older adults without dementia, based on medical history, lifestyle, social determinants of health, and laboratory tests that can be obtained in primary care settings with limited resources.

## Material and Methods

### Study design and population

This cross-sectional analysis is based on data from the Brazilian Longitudinal Study of Adult Health (ELSA-Brasil), a cohort of 15,105 active and retired civil servants from five public universities and one research institution located in six Brazilian states [São Paulo (n=5,061), Rio de Janeiro (n=1,784), Minas Gerais (n=3,115), Espírito Santo (n=1,055), Bahia (n=2,029), and Rio Grande do Sul (n=2,061)]. Baseline assessment, performed between 2008 and 2010, included participants aged between 35 and 74 years and free of dementia. Information on socio-demographics, clinical history, lifestyle, cognitive status, and occupational exposure were collected during visits to the study centers. Anthropometric measurements and laboratory and imaging tests were also obtained. The present analysis excluded 6,454 individuals younger than 50 years and 360 individuals with incomplete data on cognitive tests. The final sample included 8,291 participants. Further details of the study design and the cohort profile can be found elsewhere (17,18). The study was approved by the local ethical review boards and all participants provided informed consent.

### Outcome: global cognitive performance

A battery of neuropsychological tests was applied by trained examiners in a single session, following a fixed order. The word list memory test from the Brazilian version of the Consortium to Establish a Registry for Alzheimer's disease was used to assess memory. Participants were exposed to a list of ten unrelated printed words in three learning trials and were asked to remember the words after each trial. The sum of correctly recalled words in each trial composed the immediate memory score. After a 5-min delay, in which participants were engaged in other tasks, the examiner gave participants 60 s to recall the words and the number of correctly recalled words composed the delayed memory score. Finally, the number of correct recognitions of the 10 learned words among a list of 20 words composed the recognition memory score (19). The phonemic and semantic verbal fluency tests were applied to evaluate language and executive function. Participants were asked to name as many words starting with the letter F and as many animals as possible in 60 s. The number of correctly given distinct words and animals corresponded to the phonemic and semantic verbal fluency scores, respectively (20,21). The trail making test version B was applied to measure processing speed, visuospatial ability, and executive function. Participants were asked to draw lines connecting numbers to letters in an order that alternated between increasing numeric values and the alphabetical order. The score was composed of the total time in seconds taken to complete the task (22).

The raw scores on each cognitive test (immediate memory, delayed memory, recognition, phonemic and semantic verbal fluency, and trail making test) were transformed into z-scores by subtracting the test score of each participant from the sample mean score and dividing the difference by the sample standard deviation. Contrary to the other tests, higher scores in the trail making test indicate poorer performance. Therefore, we inverted the signal of the trail making test z-scores to reflect above-average performance when positive and below average performance when negative. A global composite z-score was calculated by averaging the z-scores on the six cognitive tests and then standardizing each participant's average using the sample mean and standard deviation. Cognitive impairment was considered when the global composite z-score was below 2 standard deviations since this cutoff is usually used to define dementia (23). The global cognitive z-score was used to define cognitive impairment since cognition is defined by performance in multiple cognitive domains.

### Predictors

Variables were selected based on the likelihood of being obtained in primary care settings. Demographic characteristic assessed were age and sex. Data on social determinants of health were education, maternal education, self-reported race, marital status, employment status, social and occupational mobility, health insurance status, family and per capita income, social class, financial problems, end of romantic relationship in the last year, role as a caregiver, and religion. Clinical conditions included diabetes, hypertension, dyslipidemia, cardiovascular, thromboembolic, renal, rheumatic, hepatic and chronic pulmonary diseases, neoplasia, headaches, bariatric surgery, hospitalization in the last year, self-reported health status, psychiatric morbidity, and use of the following classes of drugs: insulin, oral hypoglycemic agents, antihypertensives, lipid-lowering agents, platelet inhibitors, antacids, antidepressants, benzodiazepines, and ophthalmic beta-blockers. We also assessed physical measurements, such as body mass index, waist circumference, waist-hip ratio, blood pressure, and postural hypotension. Family history included coronary artery disease, stroke, sudden death, and death in the last year. Lifestyle included smoking status, alcohol consumption, physical activity, and dietary habits. Finally, laboratory tests included electrocardiogram, fasting blood glucose, glycated hemoglobin, serum creatinine, urea, sodium, potassium, transaminases, gamma glutamyl transferase, uric acid, cholesterol, triglycerides, thyroid hormones, insulin, and C-reactive protein.

### Pre-processing, data splitting, and model building

Classification models were built to predict cognitive impairment as a dichotomous variable. The code used to perform all steps to develop the models is openly available at https://github.com/labdaps/ELSACognitivePrediction. Data were divided into training (70%) and test (30%) sets, stratified according to the outcome. The training set is used to define the parameters (input variables) and hyperparameters (configurations that are external to the model and that result in the most skillful prediction) of the algorithm. The test set is new data (i.e., not used for algorithm training), used to assess the performance of the model on previously unseen data. Supplementary Table S1 shows the missing percentage for each variable before any preprocessing task. Missing variables were imputed using the MICE technique, parameterized in the training set, and applied in the test set. Because the outcome was rare and the dataset was imbalanced, we applied an oversampling technique (Synthetic Minority Oversampling Technique) in the training set prior to model training (24). Additionally, to avoid overfitting, a 10-fold cross-validation (a resampling procedure) was performed on the training set with Bayesian optimization to select hyperparameters.

We tested the most popular algorithms for the prediction of structured data: logistic regression, neural networks, and gradient boosted trees. Neural networks are ML algorithms based on the functioning of the human brain and are composed of an input layer (predictors), one or more hidden layers (where nonlinear and high-dimensional patterns are learned), and an output layer (prediction) (25). Gradient boosted trees provide a prediction result using a sequential ensemble of decision trees that are weak learners (26). These algorithms tend to have high predictive performance for structured data, especially their recent implementations like Extreme Gradient Boosting (XGBoost), Light Gradient Boosting Machine (LightGBM), and Catboost (27). While similar, they differ in a few technical instances. XGBoost adds a new term to the loss function to avoid overfitting, LightGBM grows decision trees leaf-wise, and Catboost solves the exponential growth of features by using the greedy method (28). The list of hyperparameters and their final values for the artificial neural network and the gradient boosted trees is available in Supplementary Table S2.

The main metric used to select the best model was the area under the receiver operating characteristic curve (ROC-AUC) in the test set, which measures model discrimination. A value of 0.50 is used as reference for a random estimator and a value of 1.0 is used as reference of a model with perfect discriminant ability. We evaluated the ROC-AUC values for each step of the 10-fold cross-validation technique during the training stage and no model was overfitted (Supplementary Figure S1). Additionally, we assessed the following metrics that depend on the predictive probability thresholds of the model: sensitivity (also named recall), specificity, positive predictive value (also named precision), negative predictive value, and F1-score (the harmonic mean between precision and recall). We also applied isotonic calibration in the predictive models to optimize their results. However, the calibration was inefficient, not improving the ROC-AUC metric nor the sensitivity of the model, so we opted not to maintain it.

Finally, to assess the contribution of each variable to the best performing model, we used the Shapley Additive exPlanations (SHAP) approach, which provides insight on the predictive importance of each variable and on the direction of individual values of the variable (29).

## Results

Participants' mean age was 58.3 (SD: 6.2 years), and 54.7% were female. Cognitive impairment was present in 328 individuals (4.0%). The distribution of the main sociodemographic characteristics, clinical conditions, and lifestyle according to cognitive deficit status are shown in Table 1. Participants with cognitive impairment were older, predominantly male, of black/mixed skin color, had lower education, were less active, had higher frequency of current smoking, diabetes, hypertension, and coronary artery disease, and had lower frequency of current alcohol consumption.

**Table 1:** Characteristics of participants according to cognitive deficit (n=8291).

Characteristic	Without cognitive deficit (n=7963)	With cognitive deficit (n=328)	P
Age (years), mean±SD	58.2±6.2	60.7±6.7	<0.001^a^
Female, n (%)	4417 (55.5)	119 (36.3)	<0.001^b^
Education, n (%)			<0.001^c^
<Middle school	533 (6.7)	182 (55.5)	
Middle school	680 (8.6)	57 (17.4)	
High school	2464 (30.9)	78 (23.8)	
College or higher	4286 (53.8)	11 (3.3)	
Self-reported race, n (%)			<0.001^c^
Black	1198 (15.3)	99 (31.0)	
Brown	2054 (26.2)	123 (38.4)	
White	4286 (54.6)	79 (24.7)	
Asian	218 (2.8)	11 (3.4)	
Indigenous	90 (1.1)	8 (2.5)	
Leisure-time physical activity, n (%)			<0.001^c^
Low	5979 (75.9)	272 (83.2)	
Moderate	1245 (15.8)	46 (14.1)	
High	650 (8.3)	9 (2.7)	
Smoking status, n (%)			<0.001^b^
Previous	2924 (36.7)	115 (35.2)	
Current	1033 (13.0)	67 (20.5)	
Alcohol consumption, n (%)			<0.001^b^
Previous	1574 (19.8)	109 (33.4)	
Current	5485 (69.0)	160 (49.1)	
Obesity, n (%)	1925 (24.2)	83 (25.3)	0.640^b^
Diabetes, n (%)	2055 (25.8)	128 (39.0)	<0.001^b^
Hypertension, n (%)	3635 (45.7)	204 (62.4)	<0.001^b^
Severe coronary artery disease, n (%)	314 (4.0)	20 (6.1)	0.052^b^
Global memory performance z-score, median (IQR)	0.20 (-0.60; 0.68)	-2.20 (-2.70; -1.73)	<0.001^d^
Semantic verbal fluency z-score, median (IQR)	0.06 (-0.70; 0.63)	-1.27 (-1.65; -0.89)	<0.001^d^
Trail making test z-score, median (IQR)	0.32 (-0.16; 0.61)	-2.17 (-3.66; -1.00)	<0.001^d^

IQR: interquartile range; SD: standard deviation; ^a^Student's *t*-test; ^b^Chi-squared test; ^c^Fisher exact test; ^d^Wilcoxon rank-sum test.

**Figure 1:**
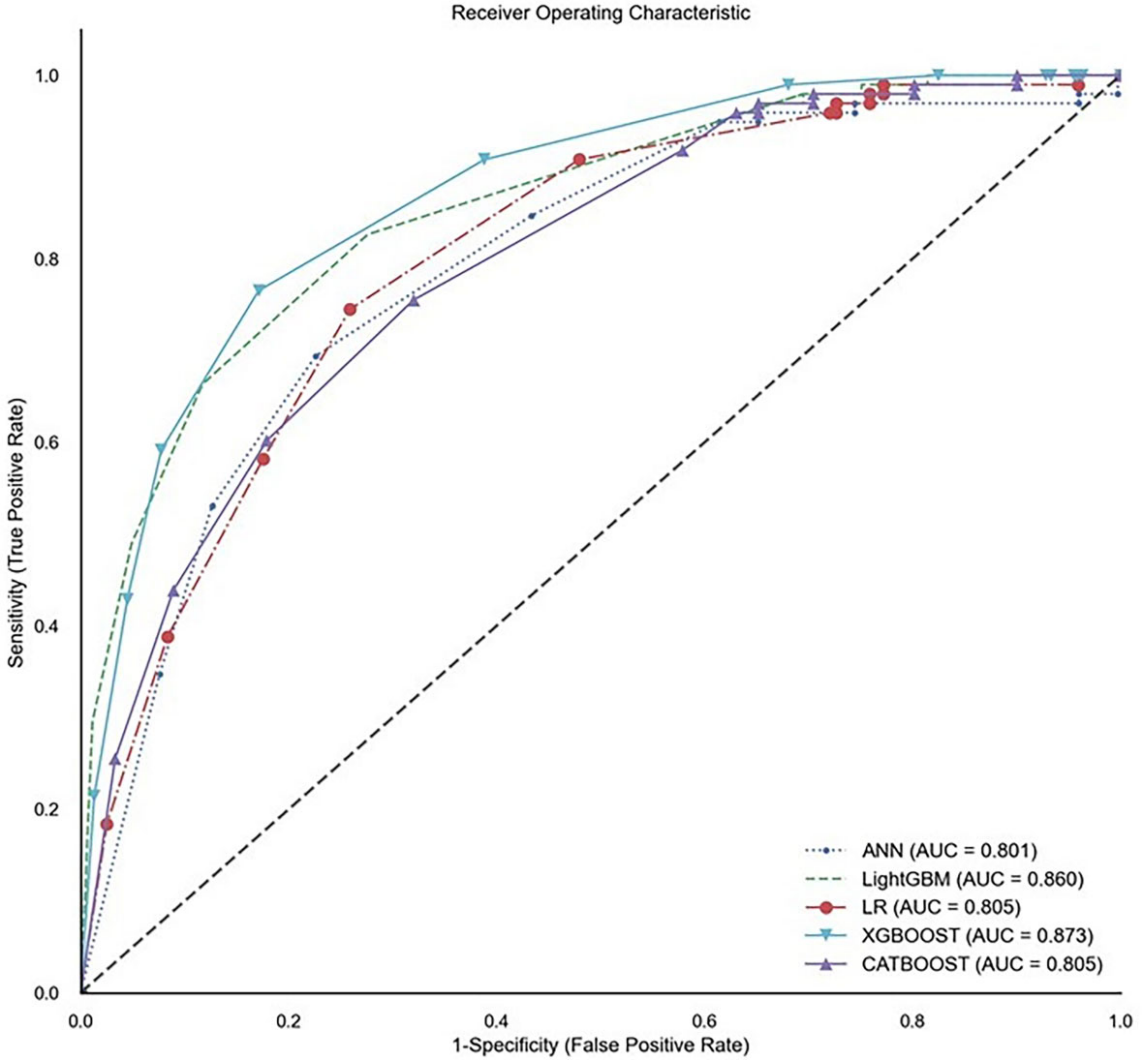
Distribution of the receiver operating characteristic curves of the different machine learning algorithms on the test set. ANN: Artificial Neural Network; AUC: area under the receiver operating characteristic curve; LightGBM: Light Gradient Boosting Machine; LR: Logistic Regression; XGBOOST: Extreme Gradient Boosting Machine.

Table 2 presents the predictive metrics results for each of the algorithms on the test set. The highest ROC-AUC and F1-score were achieved by the XGBoost (0.87 and 0.31, respectively). Among the 20% highest ranked individuals by this algorithm, 76.5% of the total individuals with cognitive impairment were included. This algorithm was able to identify negative cases with high predictive performance (specificity=0.97 and negative predictive value=0.97). The graph presenting the distribution of the ROC curves from each model is presented in Figure 1 and shows the consistent overperformance of the XGBoost and LightGBM throughout different threshold distributions.

**Table 2:** Predictive metrics results for each of the machine learning algorithms of the test set.

Model	ROC-AUC(95%CI)	Sensitivity	Specificity	PPV	NPV	F1-score	20% ranked highest^a^
XGBoost	0.873 (0.839-0.906)	0.316	0.969	0.298	0.972	0.307	76.53%
LightGBM	0.860 (0.821-0.898)	0.398	0.967	0.331	0.975	0.361	72.44%
Logistic Regression	0.805 (0.762-0.847)	0.235	0.964	0.209	0.969	0.221	61.22%
ANN	0.801 (0.755-0.845)	0.204	0.967	0.200	0.967	0.202	66.32%
Catboost	0.805 (0.762-0.847)	0.102	0.989	0.270	0.964	0.148	61.22%

ROC-AUC: area under the receiving operating characteristic curve; ANN: Artificial Neural Networks; LightGBM: Light Gradient Boosting Machine; NPV: negative predictive value; PPV: positive predictive value; XGBoost: Extreme Gradient Boosting Machine. ^a^Ranking of participants with higher probability of cognitive deficit according to each machine learning model.

**Figure 2:**
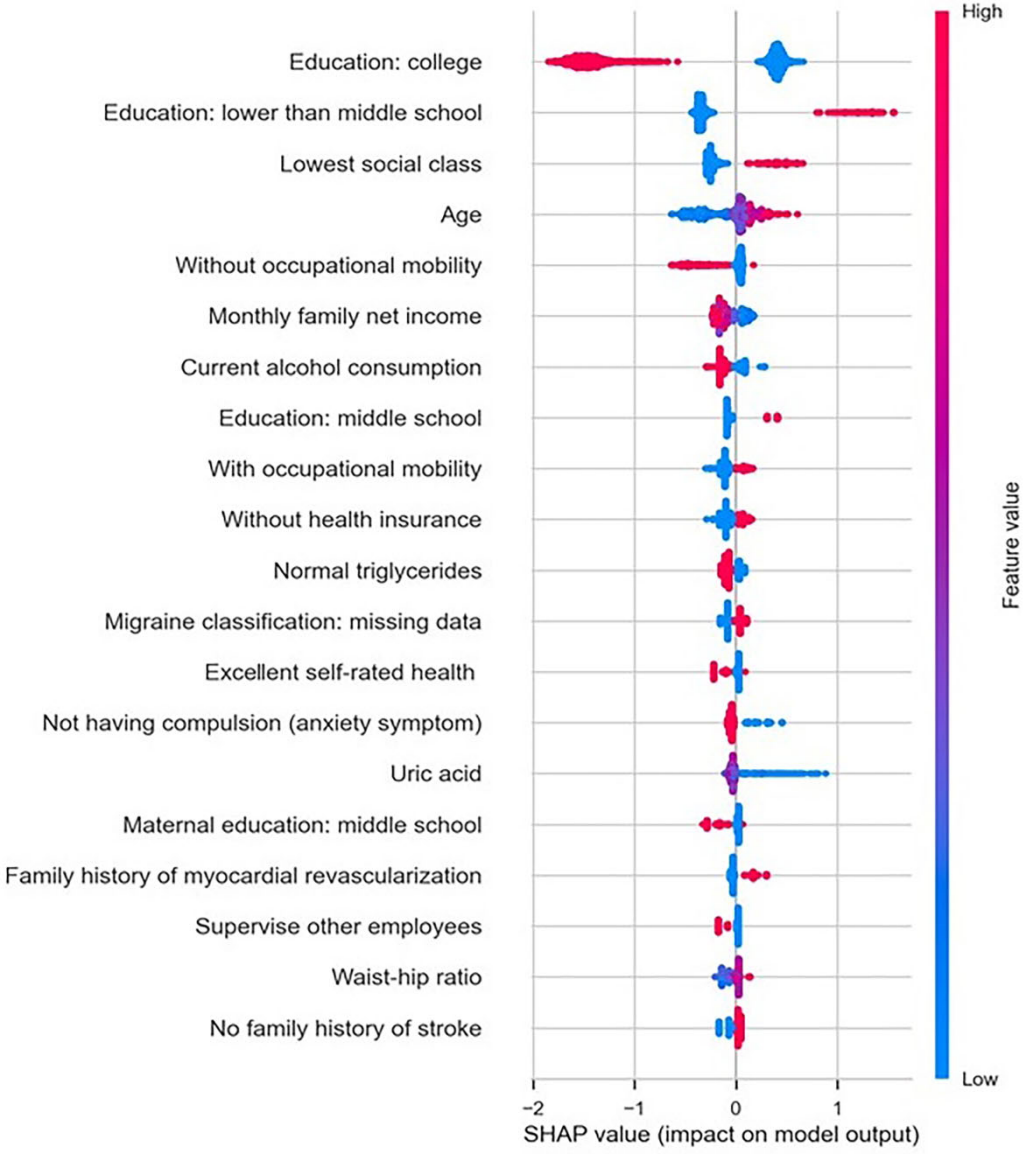
Importance of variables according to the Shapley values for the Extreme Gradient Boosting algorithm on the test set. The vertical axis lists the input variables and the horizontal axis measures their contribution in classifying cognitive deficit. The color red represents higher values and blue lower values. SHAP: Shapley Additive exPlanations.

Figure 2 presents the results of variables’ importance according to the Shapley values for the best performing algorithm (XGBoost). The most relevant predictor was education. Having at least a college degree had a negative impact on the overall risk of cognitive impairment (higher Shapley values in blue), while education lower than middle school had a positive impact on the risk of cognitive impairment (higher Shapley values in red). Other important predictors were social class, age, and lack of occupational mobility.

## Discussion

Using ML techniques, we developed and tested a predictive model for cognitive impairment based on several variables that can be assessed in primary care. The best model was developed using the XGBoost algorithm and showed high discriminant ability and specificity, which are important features to select individuals with high risk of cognitive impairment that would benefit from a detailed neuropsychological assessment.

Dementia is the main cause of disability in high-income countries, and it is among the top ten causes of disability in LMIC (30). Therefore, early detection of cognitive impairment is crucial for better health management of individual patients, as well as better allocation of resources in health systems. Currently, the diagnosis of dementia is time-consuming since it requires a detailed clinical history and physical examination, followed by an objective cognitive assessment. Dementia is secondary to neurodegenerative and cerebrovascular diseases, which are strongly related to aging. With the accelerated aging process that is taking place worldwide, performing cognitive screening in every older adult would be impractical (5,6). Therefore, it is important to develop new strategies to identify individuals at higher risk of cognitive impairment that would benefit from a full cognitive assessment.

The use of predictive models for cognitive impairment that incorporates variables that are measured in primary care settings can identify patients at risk. Some of these models used traditional statistical approaches (logistic regression, Cox proportional-hazards, and linear mixed effects) to predict dementia or cognitive decline (9-11,13,14,31,32). Most models that were based only on sociodemographic characteristics, anthropometric parameters, clinical history related to cardiovascular diseases or risk factors, lifestyle, and mental health symptoms, and did not incorporate cognitive performance as a predictor, presented fair discrimination (ROC-AUC ranging from 0.68 to 0.78) (9,11,14). The only exception was the Cox proportional-hazards model developed by Walters et al. (13), which achieved good discrimination (ROC-AUC=0.84) to predict the 5-year risk of dementia in older adults. However, neither the statistical modeling techniques, study design, or outcomes assessed in these studies could be compared to our approach.

ML models are natural extensions of traditional statistical approaches that can handle large volumes of multi-dimensional and multi-variety data, discover trends and patterns that would not be apparent to humans, are based less on human assumptions, and can improve their performance by learning from new data (16). Few ML models have been developed to predict cognition based on variables accessible in the primary care setting (15,33). Barnes et al. (33) developed and tested different classifiers to predict unrecognized dementia in community-dwelling older adults based on age, sex, medical diagnoses, healthcare utilization, vital signs, and medications. Like our study, gradient boosting achieved good discriminant ability (ROC-AUC=0.81). Na (15) developed and tested a gradient boosting machine to predict cognitive impairment in Korean older adults that achieved excellent discriminant ability (ROC-AUC=0.92). Different from our work, cognitive impairment was assessed with a screening test, the Mini-Mental State Examination, and cognitive performance was included as a feature in the model. In our study, we aimed to develop a classifier that did not rely on cognitive performance to identify individuals who would not benefit from cognitive screening and spare resources.

In the present study, sociodemographic characteristics were among the main contributors to the predictive power of the model. Education, social class, and lack of occupational mobility (which probably reflected both social class and educational level) were among the top 5 variables according to the Shapley values for the XGBoost on the testing set. Social determinants of health are strong predictors of cognitive performance, particularly in LMIC, where social inequalities are rampant (34,35). Although our aim was to identify individuals at higher risk of cognitive impairment and not to determine causal risk factors, it is well-known that socioeconomic variables, particularly education, are proxies of cognitive reserve (36).

Our ML algorithm can be used to identify individuals who are at higher risk of cognitive impairment. Seventy-six percent of the individuals with cognitive impairment were included among the 20% highest ranked individuals by the algorithm, which opens the opportunity for comprehensive screening and targeted interventions in a relatively small number of patients, which could save time and resources in busy primary care settings. Even more important for screening purposes was the fact that the algorithm also identified with high predictive performance the negative cases (specificity of 0.97 and negative predictive value of 0.97). Therefore, our algorithm will be very useful to exclude individuals with normal cognitive function, who would not benefit from clinical and neuropsychological evaluations.

Our study had some limitations. First, we performed the model validation in a test set with participants from the ELSA-Brasil, who were not included in the training set. Although this is a valid approach, the algorithm needs to be tested in other populations before it can be used in clinical practice. Second, we developed a predictive model for cognitive impairment using cross-sectional data. Future studies with longitudinal data from the ELSA-Brasil will allow the development of a predictive model for cognitive decline. Third, we did not use normative scores for cognition that would account for age and education effects. Instead, we preferred to use these variables as predictors since they have shown strong effects on cognitive performance (37). In addition, the ELSA-Brasil sample does not represent the Brazilian population since participants are public servants with higher education and income than the general Brazilian population. However, it is important to highlight that the participants' cardiovascular risk profile is similar to the general population (18). Our study strengths include the large sample size with a neuropsychological evaluation composed of tests with good sensitivity and specificity for cognitive impairment (38,39), which enabled the calculation of a global cognitive score. Other predictive models used only a screening test, such as the Mini Mental State Examination to generate a cognitive score (15,40). Moreover, data on several sociodemographic factors, clinical variables, and laboratory tests were collected, allowing to include a wide range of individual characteristics that could be assessed in primary care settings.

In conclusion, we developed and tested a predictive model for cognitive impairment using ML. The algorithm generated by the XGboost technique showed high specificity and good discriminant ability. These characteristics will allow the detection of patients who would not benefit from a cognitive assessment, facilitating the allocation of human and economic resources in primary care settings.

## Supplementary Material

Click here to view [pdf].
